# Application of GPRC5D Targeting Therapy in Relapsed Refractory Multiple Myeloma

**DOI:** 10.1002/cam4.70764

**Published:** 2025-03-17

**Authors:** Sijia Yan, Xi Ming, Rubing Zheng, Xiaojian Zhu, Yi Xiao

**Affiliations:** ^1^ Department of Hematology Tongji Hospital, Tongji Medical College, Huazhong University of Science and Technology Wuhan Hubei China

**Keywords:** bispecific T cell engagers, chimeric antigen receptor T cells, GPRC5D, multiple myeloma

## Abstract

**Background:**

As a rapidly developing therapeutic method, targeted therapy plays an important role in the treatment of multiple myeloma. In recent years, mature B cell antigen‐targeting therapy has brought new hope for patients with refractory/relapsed disease. While an increasing number of patients with relapse are exposed to this type of drug, changing the therapeutic target may be an effective strategy for patients with relapse/refractory multiple myeloma.

**Objectives:**

The expression of G protein‐coupled receptor, class C Group 5 member D (GPRC5D), on the surface of myeloma tumor cells makes it a possible target for relapse/refractory multiple myeloma therapy, and relevant studies are in progress.

**Results & Conclusions:**

The review aims to systematically summarize the current advancements in GPRC5D‐targeted therapies for multiple myeloma, thereby providing valuable insights and a foundation for future studies.

**Abbreviations**
BCMA: B‐cell maturation antigen
BsAbs: bispecific antibodies
CAR: chimeric antigen receptor
CAR‐NK: chimeric antigen receptor natural killer cells
CAR‐T: chimeric antigen receptor T cells
CR: complete response
CRS: cytokine release syndrome
CTL: cytotoxic T cells
DLT: dose‐limited toxicity
EMD: extramedullary disease
FcRH5: Fc receptor‐homolog 5
GPRC5D: G protein‐coupled receptor, class C Group 5 member D
GvHD: graft versus host disease
HLA: human leukocyte antigen
ICANS: immune effector cell‐associated neurotoxicity syndrome
MHC: major histocompatibility complex
MM: multiple myeloma
MRD: minimal residual disease
MUD‐HSCT: matched unrelated donor hematopoietic stem cell transplantation
NK: natural killer
NR: no response
ORR: overall response rate
OS: overall survival
PFS: progression‐free survival
PR: partial response
RRMM: relapsed/refractory multiple myeloma
SAE: serious adverse events
sCR: strict complete remission,
TCR: T cell redirected therapy
Tec: Teclistamab
TNFRSF17: Tumor nucleosis factor receptor superfamily 17
VGPR: very good partial remission

## 1 Introduction

Multiple myeloma (MM) is a common hematological malignancy. In recent years, with the rapid development of immunotherapy technology, therapies targeting B‐cell maturation antigen (BCMA), including the antibody coupling drug belantamab mafodotin, have shown significant advantages in MM treatment, especially in treating relapsed/refractory MM (RRMM). Chimeric antigen receptor T cells (CAR‐T) products such as idecabtagene vicleucel, ciltacabtagene autoleucel, equecabtagene autoleucel, and bispecific T‐cell engagers (BiTE) such as teclistamab [1]. Despite the success of BCMA‐targeted therapies for MM, an increasing number of patients with relapse are exposed to this class of drugs, and changing the therapeutic targets may be an effective strategy for patients with this class of RRMM [2]. Currently, studies on different targets of MM are also being carried out, including G protein‐coupled receptor, class C Group 5 member D (GPRC5D), Fc receptor‐homolog 5 (FcRH5), CD38, and CD138 [3]. Since the first identification of GPRC5D, investigations into this target have been progressing unceasingly and in depth. Concurrently, its latent capacity in treating RRMM has been continuously unearthed. This review is dedicated to elaborating on the application of GPRC5D‐targeted therapies within the realm of RRMM. It conducts a comprehensive dissection of all extant research efforts that target RRMM using GPRC5D as the focal target, encompassing both preclinical studies and clinical trials.

## 2 MM and GPRC5D

MM is a clonal plasma cell tumor with various manifestations, mainly caused by myeloma cell infiltration or abnormal monoclonal immunoglobulin elevation. It can be manifested as hypercalcemia, renal insufficiency, anemia, and bone disease, namely “CRAB” symptoms. MM can also occur in the extramedullary liver, spleen, and other tissues. The abnormal increase in immunoglobulin levels may be combined with immune paralysis, causing repeated infections, and may be combined with secondary amyloidosis and hyperviscous syndrome [4]. The RVD regimen (lenalidomide, bortezomib, and dexamethasone) is currently the most commonly used treatment regimen and has a positive effect on the initial treatment of MM patients [5]. The treatment of relapsed and refractory patients remains challenging. In recent years, the rapid development of immunotherapy has brought hope to patients with RRMMs.

**FIGURE 1:**
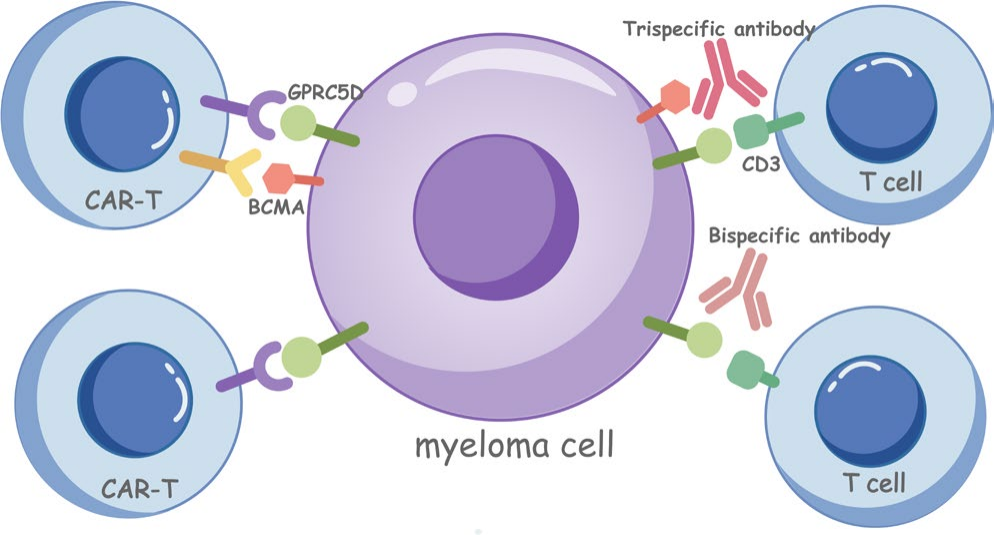
GPRC5D‐targeted therapy of relapsed/refractory multiple myeloma. BCMA, B‐cell maturation antigen; CAR‐T, chimeric antigen receptor T cells; GPRC5D, G protein‐coupled receptor, class C Group 5 member D.

GPRC5D is an orphan G protein‐coupled receptor whose ligand is unknown, and whose signaling mechanism and function in normal tissue and MM have not yet been determined [6]. Research has indicated that GPRC5D is present in both MM cells and scleratin‐producing cells [7]. Conversely, normal immune cells (T cells, B cells, NK cells, and granulocytes) exhibit low or no GPRC5D expression [8]. Notably, elevated GPRC5D levels in MM patients correlate with poorer overall survival outcomes. Therefore, GPRC5D is a promising marker for detecting tumor burden and is a reliable target for targeted therapy [9, 10] (Figure 1).

## 3 Exploration of Targeting GPRC5D Bispecific Antibody for RRMM

T cell‐conjugated bispecific antibodies (BsAbs) can bind to both tumor cell surface antigens and T cell surface CD3 so that T cells are recruited around tumor cells, activated, and degranulated, and finally achieve tumor cell clearance [11]. BsAb‐induced T‐cell‐mediated tumolysis, independent of antigen recognition by major histocompatibility complex molecules, antigen‐presenting cells, or costimulatory molecules, is an important treatment option for RRMM.

### 3.1 Talquetamab

Talquetamab, a humanized CD3 × GPRC5D bsAb, was the first BsAb to target GPRC5D in RRMM. In preclinical experiments, talquetamab induced T‐cell activation and degranulation, killing primary MM cells from newly diagnosed MM and RRMM patients. Co‐incubation with the immunomodulator pemalomide or CD38 monoclonal antibody daratumumab moderately increases the lytic effect of talquetamab on tumor cells [12]. This study provides an experimental basis for subsequent clinical trials of talquetamab alone or in combination with daratumumab or pomalidomide for the treatment of RRMM.

The Phase 1 trial MonumenTAL 1 (NCT03399799/NCT04634552) investigated the dose of talquetamab for RRMM. A total of 232 patients were administered talquetamab. At the two doses recommended for Phase 2 trials (405 μg/kg weekly and 800 μg/kg every other week), 70% and 64% of patients responded at 11.7 and 4.2 months of follow‐up, respectively, and the median duration of response was 10.2 and 7.8 months, respectively. The incidences of adverse reactions to cytokine release syndrome (CRS) were 77% and 80%, and the incidences of skin‐related events and taste disorders were 67%, 70%, 63%, and 57%, respectively [13]. The results preliminarily verified the feasibility of using talquetamab in RRMM. In the pivotal Phase 2 trial, 288 patients received talquetamab (0.4 mg/kg weekly (*n* = 143) or 0.8 mg/kg every other week (*n* = 145)). The ORR was 74% and 73%, with 59% and 57% of patients achieving a very good partial response (VGPR) or better, respectively. Of these, 51 patients who had previously received T‐cell redirected therapy (TCR) had an ORR of 63%, and 53% achieved VGPR or better responses. Common adverse reactions include CRS, skin‐related events, and taste disorders, which can be controlled [14]. Subsequent updated data analysis showed that talquetamab, targeting GPRC5D, had a sustained and robust efficacy in patients with prior exposure to TCR (primarily anti‐BCMA therapy) [15], supporting talquetamab as an effective choice for RRMM exposed to TCR‐BCMA. Based on the Phase 1/2 MonumenTAL 1 trial, talquetamab was approved for the treatment of RRMM with multiple‐wire exposure. Einsele et al. compared the efficacy of talquetamab with the real‐world physicians' choice of therapy (RWPC) for RRMM. The overall response rate (ORR), VGPR, complete response rate (CRR), progression‐free survival (PFS), and overall survival (OS) of talquetamab patients were significantly better than those of the RWPC16 patients [16], which further emphasizes the advantages of talquetamab for the treatment of RRMM.

Clinical trials on talquetamab in combination with other drugs are currently underway. Teclistamab (Tec) was the first BCMA‐targeting bispecific antibody approved for RRMM. In the ReDIRECTT‐1 clinical trial (NCT04586426), 63 patients received a combination of tec and talquetamab. The ORR was 84% in all evaluable patients and 73% in patients with evaluable extramedullary disease (EMD) at all dose levels. CR or better (≥CR) rates were 34% and 31%, respectively. At dose level 2, the ORR was 92% for all evaluable patients and 83% for the EMD‐evaluable patients. The rates of ≥CR were 31% and 33%, respectively. The median duration of response was not achieved [17]. Tec in combination with talquetamab at dose level 2 is safe and feasible for monotherapy, demonstrating that patients with RRMM can benefit from this regimen and offering them new hope. MonumenTAL 2 (NCT05050097), a Phase 1 trial of talquetamab in combination with pomadomide for the treatment of RRMM, is ongoing. Matous et al. reported that 16 and 19 patients received talquetamab 0.4 mg/kg weekly (QW) or 0.8 mg/kg every other week (Q2W), respectively. The median follow‐up periods were 11.4 and 7.7 months, respectively. The ORR in the QW and Q2W cohorts were 86.7% and 83.3%, respectively, with 60% and 44.4% of patients achieving CR and 86.7% and 77.8% of patients achieving VGPR, respectively. The median DOR and PFS were not reached, with 6‐month PFS rates of 93.3% and 88.9%, respectively [18]. Talquetamab in combination with pemadomide showed a rapid and deep response in patients with RRMM, and the safety profile was consistent with that of the monotherapy, thus supporting the use of talquetamab in combination with other agents for RRMM. However, the sample size included in this trial was small, and the follow‐up time was short; therefore, it is necessary to expand the sample size and prolong the follow‐up time for further verification. The talquetamab in combination with daratumumab clinical trial TRIMM‐2 also published data. A total of 65 RRMM patients received talquetamab combined with daratumuab, with an ORR of 78%, of which 66% achieved ≥VGPR and 45% achieved ≥CR. The median PFS was 19.4 months, and the 12‐month PFS and OS rates were 76% and 93%, respectively [19]. In the TRIMM‐2 trial, the combination of talquetamab and daratumumab induced deep and long‐lasting responses in patients with RRMM. Talquetamab, in combination with multiple drugs, has demonstrated the therapeutic advantages of bispecific antibodies, providing multiple treatment options for patients with RRMM.

### 3.2 Forimtamig

Forimtamig (RG6234) is also a CD3 × GPRC5D bispecific antibody, and unlike talquetamab, forimtamig has a new 2:1(GPRC5D:CD3) configuration, resulting in bivalent binding of GPRC5D and enhanced T‐cell redirection [20]. In a study by Jan et al., forimtamig was superior to other BsAbs in T‐cell activation, cytokine production, and proliferation, especially when co‐cultured with MM with low expression of GPRC5D; the killing effect of forimtamig was stronger than that of conventional BsAbs with a 1:1 configuration targeting GPRC5D. In in vivo experiments, forimtamig was able to eradicate tumors in xenograft mouse models. The combination of daratumumab or the triple combination of daratumumab and pomalidomide significantly enhanced the lytic effect on tumor cells [21]. Forimtamig, a BsAb capable of binding bivalently to GPRC5D, has shown unique advantages in preclinical trials, laying the foundation for subsequent clinical trials.

Iryna et al. reported the results of a Phase 1 trial of formamide for RRMM (NCT04551750) [22]. Fifty‐one patients received intravenous (IV) forimtamig and 54 received subcutaneous (SC) injections. The median follow‐up time in the IV and SC groups was 7.1 and 3.9 months, and the ORR was 71.4% and 60.4%, respectively. 57.1% and 39.6% of patients achieved ≥ VGPR, respectively. The toxicity is similar to talquetamab, including taste disturbance, dry mouth, and rash [22]. Forimtamig also demonstrated safety and efficacy in RRMM. Nevertheless, given the limited sample size and relatively brief follow‐up duration, additional research endeavors are warranted to further elucidate its potential therapeutic role and long‐term safety profile in this patient population.

### 3.3 Others

LBL‐034 is a novel T cell‐conjugated bispecific antibody that targets GPRC5D. In in vitro experiments, LBL‐034 showed cytotoxic effects on various GPRC5D cells. In the case of low GPRC5D expression, LBL‐034 could only weakly bind to CD3, indicating that LBL‐034 could only kill target cells expressing GPRC5D. In animal studies, LBL‐034 has shown good safety and tolerability [23]. Therefore, LBL‐034 is a possible choice for future GPRC5D‐targeting bispecific antibody therapy for RRMM, which needs to be validated by further clinical conversion.

Tomita et al. constructed a novel humanized bispecific monoclonal antibody, BsAb5003, targeting GPRC5D, which consists of three distinct components: a humanized anti‐CD3 single‐chain variable fragment domain, a humanized anti‐GPRC5dFAB domain, and a heterologous Fc domain with an effector mutation. In in vitro experiments, BsAb5003 induced specific cytotoxicity in GPRC5D‐positive MM cells, accompanied by T cell activation and cytokine release. In in vivo experiments, BsAb5003 redirected T cells to tumors expressing GPRC5D, inducing significant tumor growth inhibition and regression. Furthermore, combination with immunomodulators, commonly used in MM therapy, significantly enhanced T‐cell activation and cytokine production [24]. Therefore, BsAb5003 as a monotherapy or combination therapy may be an efficient or effective treatment for patients with MM and is worth further exploration.

Liu et al. developed a bispecific antibody (BR109) targeting GPRC5D. BR109 specifically triggers T cell‐mediated cytotoxicity in GPRC5D positive MM cells in vitro. Antitumor activity has also been demonstrated in xenograft mouse models of MM cell lines with human immune cell reconstitution [25].

Taken together, these preclinical studies lay a solid foundation for the practical application of monotherapy or combination therapy with bispecific antibodies targeting GPRC5D in patients with RRMM and are important prerequisites for subsequent clinical trials.

## 4 Exploration of Targeting GPRC5D CAR‐T for RRMM

As a rapidly developing adoptive cell therapy, CAR T cells play an important role in the treatment of RRMM. T cells were collected from the peripheral blood of patients' or donors' and transformed in vitro using genetic engineering technology to express the chimeric antigen receptor (CAR). These CAR‐T cells are infused into patients, specifically recognize the target antigen, proliferate rapidly, and play an antitumor role [26, 27]. At present, a variety of CAR‐T products have been put on the market, all targeting BCMA. No CAR‐T products targeting GPRC5D have been put on the market, and many clinical trials are underway.

In a preclinical experiment by Smith et al., CAR‐T cells targeting GPRC5D in vitro showed cytotoxicity toward GPRC5D‐positive MM cells. In a myeloma xenotransplantation model, CAR‐T cells targeting GPRC5D showed good amplification and antitumor activity, thus inducing tumor regression and prolonging survival time in mice. In the escape and recurrence model of the BCMA antigen, CAR‐T cells targeting GPRC5D also showed antitumor activity [28, 29]. This study demonstrates the feasibility of CAR‐T cells targeting GPRC5D in RRMM, especially in patients who have previously received anti‐BCMA therapy and provides experimental support for subsequent clinical trials.

MCARH109 is a CAR‐T with a single‐stranded variable fragment of GPRC5D derived from human B cells, a 4‐1BB costimulatory domain, and a CD3ζ signal domain. CAR‐T cell therapy targeting GPRC5D has been used to treat patients with RRMM, including those who previously received anti‐BCMA therapy. In a Phase 1 dose escalation trial (NCT04555551), 17 patients were treated with MCARH109, 47% of whom had previously received anti‐BCMA CAR‐T therapy. Ultimately, 6 patients achieved CR or better, 10 achieved very good partial remission (VGPR), and 8 had negative bone marrow minimal residual disease (MRD), with an ORR of 71%. Cytokine release syndrome (CRS) occurred in 15 patients, one of whom was more than Grade 3. One patient developed a Grade 4 immune effector cell‐associated neurotoxicity syndrome (ICANS) [30]. In addition, in this trial, there was no significant difference in the clinical response between patients who had previously received anti‐BCMA treatment and those who had not. Therefore, this study supports the efficacy of MCARH109 in RRMM and provides a new treatment strategy for patients with recurrent BCMA. POLARIS is a Phase I clinical trial (NCT05016778) of GPRC5D targeting CAR‐T(OriCAR‐017) in RRMM. Among the 10 patients who received OriCAR‐017 infusion, 6 achieved strict complete remission (sCR), 4 achieved VGPR, and the ORR was 100%. All patients tested negative for MRD at Day 28. In terms of safety, no dose‐limited toxicity (DLT), serious adverse events (SAE), or deaths were reported [31]. This experiment supported the effectiveness and safety of CAR‐T cells targeting GPRC5D in RRMM. Clinical trial CC‐95266‐MM‐001(NCT04674813) evaluated the efficacy of BMS‐986393, a CAR‐T cell targeting GPRC5D, in RRMM. The ORR and CRR of the 70 patients were 86% and 38%, respectively. Among the 32 refractory patients who previously received BCMA‐targeted therapy, the ORR was 85% and 46% [32]. The safety and effectiveness of BMS‐986393 in RRMM were preliminarily supported in the first‐phase experiment, which provided data support for the second‐phase experiment with an enlarged sample size.

In China, Xia et al. launched a Phase II clinical trial targeting GPRC5D CAR‐T cells to treat RRMM. In this trial, 33 patients received an infusion of anti‐GPRC 5CAR‐T. At a median follow‐up of 5.2 months, the ORR was 91%, including 11, 10, 4 cases of VGPR and 5 cases of sCR, CR, VGPR, and PR, respectively. Among them, 9 patients who had previously received anti‐BCMA CAR‐T therapy achieved PR or better. CRS occurred in 25 patients, all of whom were Grade 1 or 2. ICANS appeared in 3 patients [33]. In this clinical trial, anti‐GPRC 5CAR‐T showed encouraging safety and effectiveness, especially in patients who failed anti‐BCMA CAR‐T treatment.

Li et al. also developed a GPRC5D CAR‐T whose CAR structure consists of a single‐chain variable fragment, a 4‐1BB co‐stimulatory domain, and CD3ξ binding to GPRC5D. A total of 7 patients with RRMM received this autologous CAR‐T infusion, 3 of whom had previously received BCMA‐targeted CAR‐T therapy. At the time of efficacy evaluation, the ORR was 85.7%, including 3 CR or better, 3 PR, and 1 no response. Six patients developed CRS, all of which were Grade 1 or Grade 2. No patients developed ICANS [34]. Therefore, this GPRC5D‐targeted CAR‐T is also a possible direction for the treatment of RRMM, and further studies with larger sample sizes are needed.

The results of several clinical trials (Table 1) show that CAR‐T cells targeting GPRC5D are safe and effective for patients with RRMM. Whether patients with RRMM have received anti‐BCMA therapy in the past, especially CAR‐T therapy targeting BCMA, CAR‐T against GPRC5D is a potential choice, which brings new hope to these patients. At present, several clinical trials targeting GPRC5D CAR‐T cells for the treatment of RRMM are underway, and their results are expected to be announced.

**TABLE 1:** Existing results of CAR‐T targeting GPRC5D in the treatment of relapsed/refractory multiple myeloma.

CAR‐T	Phase	n	ORR	CRR	PR	CRS	ICANS	Previously received BCMA‐targeted therapy
MCARH109	I	17	71%	35%	71%	88%	6%	59%
OriCAR‐017	I	10	100%	60%	40%	100%	0	50%
BMS‐986393	I	70	86%	38%	/	84%	11%	46%
GPRC5D CAR‐T	II	33	91%	64%	27%	76%	9%	27%
GPRC5D CAR‐T	I	7	85.7%	42.8%	42.8%	85.7%	0	42.8%

Abbreviations: BCMA, B‐cell maturation antigen; CAR‐T, chimeric antigen receptor T cells; CRR, complete response rate; CRS, cytokines release syndrome; ICANS, immune effector cell‐associated neurotoxicity syndrome; ORR, overall response rate; PR, partial response.

However, CAR‐T cells targeting GPRC5D in RRMM also pose the risk of recurrence. Mi et al. found that six patients with RRMM relapsed after CAR‐T cell infusion in a Phase I study of MCARH109. The expression of GPRC5D mRNA and protein decreased or was absent in all patients when the disease recurred, and one patient lost the GPRC5D bi‐allelic gene when the disease recurred [35]. Antigen escape is an important reason for the recurrence of GPRC5D‐targeted CAR‐T cells. Previous studies have shown that dual‐targeted CAR‐T cells have a better antitumor effect than single‐targeted CAR‐T [36]. Zhou et al. reported, for the first time, a clinical trial (NCT05509530) of CAR‐T cells with double targeting of BCMA/GPRC5D for the treatment of RRMM. In their study, 21 patients received anti‐BCMA/GPRC5D CAR T‐cell infusion. After a median follow‐up of 5.8 months, the total remission rate was 86%; 13 patients (62%) were in complete remission or better and 17 patients (81%) were negative for measurable residual lesions. The 10‐month progression‐free survival rate was 67%. Cytokine release syndrome occurred in 15 patients, all of whom were Grade 1–2; one patient developed immune effector cell‐related toxic syndrome [37]. Anti‐BCMA/GPRC5D CAR‐T cells are effective and safe for the treatment of patients with RRMM. Bispecific CAR‐T cells can target MM tumor cells more widely and reduce the risk of antigen escape; therefore, they are one of the possible choices for single‐targeted CAR‐T cells to treat relapsed patients. However, the sample size included in this study was small, and the follow‐up period was short. Therefore, randomized controlled trials with longer follow‐up periods and larger sample sizes are required to evaluate and compare their long‐term results [38].

## 5 Exploration of Targeting GPRC5D CAR‐NK for RRMM

Compared with CAR‐T cells, CAR‐NK cells have been widely used in the treatment of malignant tumors because of their low incidence of graft‐versus‐host disease and lower toxicity [39]. FT555 is a GPRC5D‐targeting CAR‐NK derived from induced pluripotent stem cells. In vitro, FT555 is a GPRC5D‐targeting CAR NK cell line derived from iPSCs. In vitro, FT555 showed sustained tumor‐specific activity against GPRC5D and demonstrated strong killing kinetics and tumor clearance ability in a xenograft model to prolong the survival of mice. In combination with daratumumab, the persistence of FT555 was enhanced, resulting in enhanced inhibition of tumor growth and significantly prolonged survival [40]. In a study by Cao et al., CAR‐NK targeting both BCMA and GPRC5D had a killing effect on MM cells, and compared with CAR‐T targeting BCMA alone, BCMA/GPRC5D dual‐targeted CAR‐NK cells effectively lysed BCMA‐negative MM cells, improved the survival rate of MM xenografts, and reduced tumor recurrence [41]. Dual‐targeting CAR‐NK is also a possible choice for patients with RRMM, especially those who fail anti‐BCMA therapy, and further preclinical experiments and clinical transformation are expected.

## 6 Challenges of Targeting GPRC5D in the Treatment of RRMM

### 6.1 Antigen Escape

Although GPRC5D‐targeted therapies have demonstrated favorable therapeutic outcomes in patients with RRMM, a subset of patients still exhibits relapse post‐treatment. Such acquired resistance is likely to be mediated by a multitude of factors, including antigen escape, disease burden, and T‐cell exhaustion [42]. Antigen escape is the most common problem in targeted therapy and is an important cause of treatment failure. GPRC5D expression is often reduced or absent in patients who relapse after GPRC5D‐targeted therap [43]. Lee et al. found that the orphan gene encoding GPRC5D is more prone to mutation than tumor necrosis factor receptor superfamily 17 (TNFRSF17) encoding BCMA, including the deletion of two alleles or a single copy number with multiple GPRC5D mutation events (single‐nucleotide deletion, large frame mutation, and balanced translocation) [44].

The simultaneous use of multiple bispecific antigens prevents the antigens from escaping [17]. Trispecific antibodies targeting two or more different tumor‐associated antigens simultaneously and multi‐targeted CAR‐T cells are also important for preventing recurrence caused by antigen escape, which can kill tumor cells more specifically and reduce the toxicity of extratumor targeting [45]. JNJ‐79635322 is a trispecific antigen that targets BCMA, GPRC5D, and CD3. In vitro, JNJ‐79635322 was highly cytotoxic to many MM cell lines and effectively depleted double‐positive and single‐positive target cells of BCMA and GPRC5D. In xenotransplantation mice, JNJ‐79635322 showed strong antitumor activity in a model expressing a single and double target, respectively [46]. A Phase I dose escalation trial (NCT05652335) of JNJ‐79635322 for the treatment of RRMM is ongoing, and the results are expected to be published. Furthermore, the ongoing clinical trial of three target‐specific antigens for the treatment of RRMM is IBI3003 (NCT06083207), which provides a preventive scheme for antigen escape in tumor treatment.

### 6.2 Infection

Post‐treatment infection is a common problem associated with immunotherapy. Jourdes et al. reported that 51% of patients treated with bispecific antibodies against GPRC5D were infected, which was lower than the 73% of patients treated with anti‐BCMA [47]. When the GPRC5D bsAb was combined with daratumumab and/or pomalidomide, the risk of infection increased. Pre‐treatment hypoalbuminemia and lymphopenia are important risk factors for infection [11, 48]. Most infections are caused by common pathogens such as respiratory viruses and *Streptococcus pneumoniae* . At present, in the practical recommendation of monitoring and preventing infection, it is strongly recommended to use antibacterial therapy routinely in the first month of treatment to prevent infection [49]. Intravenous immunoglobulin is also an important means of preventing infection during bispecific antibody and CAR‐T cell therapy. Prevention of herpes virus and pneumocystis pneumonia should also be considered for these patients with an increased risk of infection [11].

### 6.3 Off‐Tumor Toxicities

Off‐tumor toxicities mean that tumor cells kill target tumor cells as well as normal cells expressing the target antigen, which is a common problem in targeted therapy. GPRC5D is expressed not only on the surface of MM tumor cells but also in cells producing hard keratin [7].

#### 6.3.1 Cutaneous and Mucosal Toxicities

Cutaneous and mucosal toxicities frequently manifest during the treatment targeting GPRC5D. Specifically, skin toxicities are observed in all 5 patients administered talquetamab, presenting as Sjogren's syndrome, hand‐foot syndrome, maculopapulosis, and inflammatory palmoplantar keratosis with fissure formation [50]. Moreover, 86% of patients treated with bispecific antibodies exhibit analogous symptoms [51], and the phenomenon also evident in those receiving GPRC5D‐targeted CAR‐T cell therapy. Regarding nail toxicities, conditions like onychomadesis/Beau's lines and dystrophia unguium are noted in MM patients treated with talquetamab. Oral mucosal alterations include symptoms such as dry mouth, taste disorders, and intraoral pain [31, 32, 37]. Cutaneous‐directed therapeutic interventions, such as the topical application of corticosteroids and emollients, can effectively ameliorate skin‐related symptoms.

#### 6.3.2 Cerebellar Dysfunction

Cerebellar disorder is a characteristic side effect of CAR‐T cells targeting GPRC5D for the treatment of RRMM. In Mailankody's report, 2 patients developed clinical manifestations of cerebellar dysfunction such as gait instability, ataxia, and dysarthria after CAR‐T infusion. Although they received a variety of treatments including oral glucocorticoids, high‐dose methylprednisolone, intravenous immunoglobulin, and meclozine, their Grade 3 cerebellar dysfunction persisted during the follow‐up periods of 7.7 months and 10.8 months [30]. It has been found that GPRC5D is expressed in the inferior olivary nucleus of the medulla oblongata, which is responsible for transmitting motor and sensory signals to the cerebellum and regulating motor coordination [52]. This may be related to the occurrence of cerebellar dysfunction.

### 6.4 Others

CRS and ICANS are common adverse reactions to GPRC5D treatment. The former is caused by an increase in inflammatory markers and cytokines in the serum during treatment and is characterized by fever, muscle spasms, and fatigue [53]. The latter may be related to many factors such as cytokine‐mediated toxicity, endothelial cell activation, and blood–brain barrier damage during treatment, which may manifest as aphasia, changes in consciousness levels, cognitive impairment, motor weakness, seizures, and brain edema [54, 55]. Symptomatic support therapy is the main treatment for both, while steroid hormones are the most used drugs. Tocilizumab is also used in the treatment of CRS [56] (Table 2).

**TABLE 2:** Challenges of targeting GPRC5D in the treatment of relapsed/refractory multiple myeloma.

Challenges	Solutions	Efficacy
Antigen escape	Combination of multiple bispecific antigens; Trispecific antibodies targeting two or more different tumor‐associated antigens	Effective
Infections	Antibacterial therapy; Intravenous immunoglobulin	Pending verification
Off‐tumor toxicities	Skin‐oriented therapy, such as local steroid hormones and humectants; Oral glucocorticoid, high‐dose methylprednisolone, intravenous immunoglobulin	The local treatment for skin toxicity is effective, whereas the treatment for neurological toxicity shows limited efficacy.
Others: cytokine release syndrome and immune effector cell‐associated neurotoxicity syndrome	Symptomatic support therapy and tocilizumab for cytokine release syndrome	Effective

## 7 Conclusion

In recent years, targeted therapies have achieved remarkable progress in the treatment of hematological malignancies. MM, being one of the most prevalent hematological malignancies, has seen therapeutic strategies targeting BCMA reach a relatively mature stage. The therapeutic landscape of MM is further expanding with the emergence of GPRC5D as a promising target. The application prospects of therapeutic methods targeting GPRC5D in MM treatment are vast. Large‐scale, multicenter clinical studies are warranted to systematically evaluate the synergistic effects of different treatment modalities and drug combinations, with the aim of identifying the most efficacious treatment regimens. Concurrently, long‐term follow‐up studies are indispensable, necessitating vigilant monitoring of long‐term disease control, risk of relapse, and changes in quality of life post‐treatment. Moreover, the active pursuit of novel combination therapy targets is of great significance. Through the synergistic action of multiple targets, there is the potential to develop more efficient and safer comprehensive treatment plans, thereby offering MM patients more favorable treatment outcomes.

Overall, as more clinical trials are conducted and data accumulate (Table 3), GPRC5D‐targeted therapy is anticipated to become a significant breakthrough in the field of MM treatment [57]. Future research should continue to explore superior MM treatment strategies to maximize patient benefits.

**TABLE 3:** Clinical trials of therapies targeting GPRC5D.

	Targets	Products	Trails	Status
CAR‐T	GPRC5D	CAR‐GPRC5D	NCT05219721	Unknown status
		CT071	NCT06333509	Not yet recruiting
		CAR‐GPRC5D	NCT05759793	Recruiting
		DeepTag‐GPRC5D CAR‐T	NCT06084962	Recruiting
		GPRC5D‐CAR‐T	NCT05016778	Active, not recruiting
		Anti‐GPRC5D CAR‐T	NCT05739188	Recruiting
		Anti‐GPRC5D CAR‐T	NCT05749133	Recruiting
		BMS‐986393	NCT06297226/NCT06121843	Recruiting
		MCARH109	NCT04555551	Active, not recruiting
		OriCAR‐017	NCT06182696/NCT06271252	Recruiting
		RD140	NCT06655519	Not yet recruiting
	BCMA+GPRC5D	Anti‐BCMA/GPRC5D CAR‐T	NCT05509530	Recruiting
		Anti‐BCMA‐GPRC5D CAR‐T	NCT06515262	Recruiting
		BMCA and GPRC5D dual target CAR‐T cells	NCT05325801	Not yet recruiting
		BCMA‐GPRC5D CAR‐T‐cells	NCT05998928	Recruiting
		BCMA/GPRC5D double CAR‐T	NCT06068400	Recruiting
		MCARH109 and MCARH125	NCT05431608	Recruiting
		BCMA‐GPRC5D CAR‐T	NCT06644443	Recruiting
	GPRC5D + CD19	GPRC5D‐CD19 CAR T	NCT06298266	Not yet recruiting
CAR‐NK	BCMA+GPRC5D	Anti‐BCMA/GPRC5D Bispecific CAR‐NK Cells (ACT‐001)	NCT06594211	Not yet recruiting
Monoclonal antibody	GPRC5D	SAR446523	NCT06630806	Not yet recruiting
Bispecific antibodies	GPRC5D + CD3	Dermo‐cosmetic products	NCT06418750	Not yet recruiting
		JNJ‐64407564	NCT04773522	Active, not recruiting
		Talquetamab	NCT04634552/NCT03399799	Recruiting
Trispecific antibodies	GPRC5D + BCMA+CD3	SIM0500	NCT06375044	Recruiting
		IBI3003	NCT06083207	Recruiting
		JNJ‐79635322	NCT05652335	Recruiting

*Note:* All data comes from https://clinicaltrials.gov/.

Abbreviations: BCMA, B‐cell maturation antigen; CAR‐NK, chimeric antigen receptor natural killer cells; CAR‐T, chimeric antigen receptor T cells; GPRC5D, G protein‐coupled receptor, class C Group 5 member D.

## Author Contributions

**Sijia Yan:** writing – original draft. **Xi Ming:** writing – original draft. **Rubing Zheng:** writing – original draft. **Xiaojian Zhu:** writing – review and editing. **Yi Xiao:** funding acquisition, writing – review and editing.

## Ethics Statement

The authors have nothing to report.

## Consent

The authors have nothing to report.

## Conflicts of Interest

The authors declare no conflicts of interest.

## Data Availability

No datasets were generated or analyzed during the current study.
